# Characteristics and risk factors associated with critical illness in pediatric COVID-19

**DOI:** 10.1186/s13613-020-00790-5

**Published:** 2020-12-19

**Authors:** Grace Fisler, Stephanie M. Izard, Sareen Shah, Deirdre Lewis, Mundeep K. Kainth, Stefan H. F. Hagmann, Joshua A. Belfer, Lance M. Feld, Fiore Mastroianni, Charlotte L. Kvasnovsky, Christine A. Capone, James Schneider, Todd Sweberg, Charles Schleien, Matthew D. Taylor, the Northwell COVID-19 Research Consortium

**Affiliations:** 1 Division of Pediatric Critical Care Medicine, Steven and Alexandra Cohen Children’s Medical Center of New York, Northwell Health, Donald and Barbara Zucker School of Medicine At Hofstra/Northwell, 269-01 76th Ave, New Hyde Park, NY 11040 USA; 2 Department of Pediatrics, Steven and Alexandra Cohen Children’s Medical Center of New York, Northwell Health, Donald and Barbara Zucker School of Medicine At Hofstra/Northwell, New Hyde Park, NY 11040 USA; 3 Center for Health Innovations and Outcomes Research, Feinstein Institutes for Medical Research, Northwell Health, Manhasset, NY 11030 USA; 4 Division of Pediatric Infectious Diseases, Steven and Alexandra Cohen Children’s Medical Center, Northwell Health, Donald and Barbara Zucker School of Medicine At Hofstra/Northwell, New Hyde Park, NY 11040 USA; 5 Division of Pulmonary, Critical Care, and Sleep Medicine, Northwell Health, Donald and Barbara Zucker School of Medicine At Hofstra/Northwell, New Hyde Park, NY 11040 USA; 6 Division of Pediatric Surgery, Steven and Alexandra Cohen Children’s Medical Center of New York, Northwell Health, Donald and Barbara Zucker School of Medicine At Hofstra/Northwell, New Hyde Park, NY 11040 USA

**Keywords:** COVID-19, CRP, Organ dysfunction, Pediatrics

## Abstract

**Background:**

While much has been reported regarding the clinical course of COVID-19 in children, little is known regarding factors associated with organ dysfunction in pediatric COVID-19. We describe critical illness in pediatric patients with active COVID-19 and identify factors associated with PICU admission and organ dysfunction. This is a retrospective chart review of 77 pediatric patients age 1 day to 21 years admitted to two New York City pediatric hospitals within the Northwell Health system between February 1 and April 24, 2020 with PCR + SARS-CoV-2. Descriptive statistics were used to describe the hospital course and laboratory results and bivariate comparisons were performed on variables to determine differences.

**Results:**

Forty-seven patients (61%) were admitted to the general pediatric floor and thirty (39%) to the PICU. The majority (97%, n = 75) survived to discharge, 1.3% (*n* = 1) remain admitted, and 1.3% (*n* = 1) died. Common indications for PICU admission included hypoxia (50%), hemodynamic instability (20%), diabetic ketoacidosis (6.7%), mediastinal mass (6.7%), apnea (6.7%), acute chest syndrome in sickle cell disease (6.7%), and cardiac dysfunction (6.7%). Of PICU patients, 46.7% experienced any significant organ dysfunction (pSOFA >  = 2) during admission. Patients aged 12 years or greater were more likely to be admitted to a PICU compared to younger patients (*p* = 0.015). Presence of an underlying comorbidity was not associated with need for PICU admission (*p* = 0.227) or organ dysfunction (*p* = 0.87). Initial white blood cell count (WBC), platelet count, and ferritin were not associated with need for PICU admission. Initial C-reactive protein was associated with both need for PICU admission (*p* = 0.005) and presence of organ dysfunction (*p* = 0.001). Initial WBC and presenting thrombocytopenia were associated with organ dysfunction (*p* = 0.034 and *p* = 0.003, respectively).

**Conclusions:**

Age over 12 years and initial CRP were associated with need for PICU admission in COVID-19. Organ dysfunction was associated with elevated admission CRP, elevated WBC, and thrombocytopenia. These factors may be useful in determining risk for critical illness and organ dysfunction in pediatric COVID-19.

## Background

The SARS-CoV-2 virus has overwhelmed healthcare systems around the world [1, 2]. Disease presentation varies from asymptomatic infections identified through contact tracing to fulminant respiratory failure refractory to conventional therapies. Initial data concluded that children experienced predominantly mild illness with only a handful requiring ICU admission and mechanical ventilation [3–8]. Later data identified serious disease in a small subset of children, but concluded the vast majority experience mild infection [9, 10].

The first study on critically ill children with COVID-19 described 48 patients admitted to 14 pediatric intensive care units (PICU) in the U.S. Over 80% of patients had underlying medical conditions and two died [11]. These data are congruent with another multicenter study that described a high rate of comorbidities and adolescent age in pediatric patients requiring PICU admission for COVID-19 [12]. Need for mechanical ventilation in children with COVID-19 has been associated with elevated CRP in one single-center study [13].

The clinical course of pediatric COVID-19 has been described in many studies, however there is a paucity of literature examining factors that contribute to organ dysfunction in children [14–16]. In this study, we set out to determine factors associated with PICU admission and acute organ dysfunction in COVID-19 patients. We compare the characteristics between three groups of pediatric patients admitted with COVID-19 infection: patients not requiring PICU care, patients admitted to the PICU with minimal or no organ dysfunction, and patients admitted to the PICU with organ dysfunction.

## Materials and methods

We conducted a retrospective cohort study of all patients under 21 years of age (*n* = 77) admitted with COVID-19 to either of our two hospitals that offer pediatric intensive care from February 1, 2020 through April 24, 2020, during the height of the pandemic in the New York City area. Data collection closed on June 20, 2020. The dataset was the same used in an epidemiology study examining admitting symptoms, but encompassed a longer study period and included patients at our second hospital [17]. The study was approved by the Northwell Health System Institutional Review Board (#20-0318-CCMC) and granted a waiver of informed consent.

Cases were identified by medical record documentation of COVID-19 infection in any patient admitted to our pediatrics service. Data collected included demographics, indication for admission, BMI, presence of pre-existing conditions, hospital outcomes, and PELOD-2 and pSOFA scores [18, 19]. Lab values were collected within 24 h of admission and included white blood cell count (WBC), platelet count, C-reactive protein (CRP), and ferritin. ICU interventions were also recorded and analyzed. Pediatric acute respiratory distress syndrome (pARDS) was defined using non-invasive measurements of oxygenation [20].

Data were collected and managed using REDCap electronic data capture tools hosted at Northwell Health [21, 22]. All analyses were performed using SAS Studio version 3.8 with a *p *value < 0.05 considered statistically significant.

We identified several possible factors associated with ICU admission and organ dysfunction a priori*.* These were extrapolated from adult COVID-19 and pediatric multiorgan dysfunction literature. Factors suspected to be associated with severe disease included older age, presence of pre-existing comorbidity, increased BMI, initial WBC, platelet count, CRP, and ferritin [23–25].

Age was categorized into < 60 days, 60 days to < 5 years, 5 to < 12 years, or >  = 12 years, based on sample distribution and clinical relevance. For inferential analysis, age categories were collapsed into < 12 years versus >  = 12 years. BMI was converted into BMI percentile for those < 20 years old [26]. Patients in < 5th percentile were categorized as underweight, 5–85th as normal weight, 85–95th as overweight, and > 95th as obese. For patients aged 20–21 years, those with BMI < 18.5 were considered underweight, 18.5 to 25 as normal weight, 25 to 30 as overweight, and > 30 as obese[27]. Patients < 2 years were excluded in BMI categorization. For these analyses, categorized BMI was dichotomized into (1) underweight or normal weight and (2) overweight or obese. Need for respiratory support was defined by need for high-flow nasal cannula (HFNC) above 4 L per minute, non-invasive positive pressure ventilation (NIPPV), or invasive mechanical ventilation (MV). Patients with missing data were excluded from individual inferential analyses. Outcomes of interest included ICU admission during hospital stay (yes versus no) and organ dysfunction (yes versus no).

All variables were summarized descriptively within the overall sample and the subgroups of floor only, ICU admission for organ dysfunction and for other reasons. The Chi-square or Fisher’s exact test was used for categorical variables of interest such as any significant differences across ICU admission and organ dysfunction outcomes. The Wilcoxon rank sum test was used for continuous variables.

The pSOFA score is an adaptation of the adult sequential organ function assessment (SOFA), a part of the current adult definition of sepsis [18, 28]. An increase in SOFA score of >  = 2 is associated with a mortality of 10% in adult patients [29]. We utilized an increase in pSOFA score of >  = 2 to define significant organ dysfunction in pediatric patients. We screened for baseline organ dysfunction when information was available and adjusted the pSOFA score to reflect the increase in pSOFA score from baseline. We also examined the PELOD-2 (Pediatric Logistic Organ Dysfunction) score as a second measure.

## Results

### *General characteristics of patients admitted for pediatric COVID-19 (Table **1**)*

**Table 1:** Demographic data and comorbidities

	Total cohort (*N* = 77)	Floor patients (*N* = 47)	PICU low acuity (*n* = 16)^a^	PICU organ dysfunction (*n* = 14)^b^
Demographic data				
Age (*n*, %)				
Age < 60 days	20 (26)	16 (34)	3 (19)	1 (7)
Age 60 days to < 5 years	10 (13)	6 (13)	2 (13)	2 (14)
Age 5 years to < 12 years	9 (12)	7 (15)	0	2 (14)
Age > = 12 years	38 (49)	18 (38)	11 (69)	9 (64)
Female sex (*n*, %)	40 (53)	23 (49)	9 (56)	8 (57)
BMI (percentile, *n*, %)^c,d^				
Underweight (< 5th percentile)	2 (4)	2 (8)	0	0
Normal weight (5–85th percentile)	26 (58)	13 (52)	7 (78)	6 (55)
Overweight (85–95th percentile)	5 (11)	3 (12)	1 (11)	1 (9)
Obese (> 95th percentile)	12 (27)	7 (28)	1 (11)	4 (36)
Race (*n*, %)				
Black	22 (29)	10 (21)	7 (44)	5 (38)
White	15 (19)	12 (26)	3 (19)	0
Asian	9 (12)	5 (11)	1 (6)	3 (21)
Multiracial/other	28 (36)	17 (36)	5 (31)	6 (43)
Unknown	3 (4)	3 (6)	0	0
Ethnicity (*n*, %)				
Hispanic	18 (23)	9 (19)	3 (19)	6 (43)
Non-Hispanic	55 (71)	37 (79)	11 (69)	7 (50)
Unknown	4 (5)	1 (2)	2 (13)	1 (7)
Comorbidities (*n*, %)				
Prematurity	7 (9)	6 (13)	1 (6)	0
Respiratory disease	16	8 (17)	5	3 (21)
Congenital heart disease	5 (6)	2 (4)	2 (13)	1 (7)
Diabetes mellitus	5 (6)	2 (4)	2 (13)	1 (7)
Immunosuppression	9 (12)	6 (13)	2 (13)	1 (7)
Autoimmune disorder	2 (3)	1 (2)	1 (6)	0
Bone marrow transplant	2 (3)	1 (2)	0	1 (2)
Kidney disease	1 (1)	1 (2)	0	0
Cancer	5 (6)	3 (6)	1 (6)	1 (7)
Genetic disorder	7 (9)	2 (4)	1 (6)	4 (29)

^a^PICU low acuity defined as pSOFA score < 2

^b^PICU organ dysfunction defined as pSOFA score >  = 2

^c^BMI category presented for 45 patients (25 floor, 11 ICU organ days, 9 other). 25 missing under 2 years old, 7 missing older than 2 years

^d^BMI category for those 20–21 classified as underweight < 18.5, normal 18.5–25, overweight 25–30, obese > 30

Seventy-seven patients met the inclusion criteria. All patients were COVID-19 PCR positive by nasopharyngeal swab. All included patients had primary COVID-19 infection and none were diagnosed with Multisystem Inflammatory Syndrome in Children (MIS-C) as the data collection period encompassed the time of the initial, primary outbreak of COVID-19 in the region. Of 77 patients (mean age = 9.5 years), 71 were admitted to the tertiary children’s medical center and six to the satellite hospital. Thirty (39%) were admitted to a PICU and 47 (61%) to a general inpatient ward. No differences were noted in gender, race, ethnicity, or general pre-existing comorbidities.

### Characterization of organ dysfunction associated with pediatric COVID-19

Of 30 ICU patients, 50% presented with hypoxia and 20% with hemodynamic instability. Other notable admission indications are in Table 2. Within ± four hours of PICU admission, 63.3% experienced documented hypoxia (defined as SpO2 < 90% on room air) and 10% required vasoactive medications. Two patients (6.7% of PICU cohort) required renal replacement therapy. There were no patients with underlying chronic kidney disease in the PICU cohorts. Of PICU patients, 46.7% experienced organ dysfunction (Table 2). Specific organ system dysfunction, defined using pSOFA score >  = 1 for organ system category, showed a predominance of cardiovascular and respiratory failure (Table 2).

**Table 2:** PICU admittance and characteristics

Reason(s) for PICU admission *n* (%)	Total PICU cohort (*N* = 30)
Hypoxia	15 (50)
Hemodynamic instability	6 (20)
DKA	2 (7)
Cardiac dysfunction	2 (7)
Mediastinal mass	2 (7)
Apnea	2 (7)
Acute chest syndrome in sickle cell disease	2 (7)
Other	6 (20)
Outcomes	
Length of ICU stay, days [mean (median, IQR)]^a^	8.8 (3.6, 2.5–14.7)
ICU discharge (*n*, %)	29 (97)
Deceased (*n*, %)	1 (3)

Respiratory failure	Intubated cohort (*N* = 8)
OSI at intubation [mean (median, IQR)]	14 (15, 5.5–20)
Maximum PEEP [mean (median)]	12 (13.5)
Ventilator-free days [mean (median, IQR)]	14 (15, 13–23)

PICU admission characteristics	Total cohort (*N* = 30)	PICU low acuity (*n* = 16)	PICU organ dysfunction (*n* = 14)
pSOFA [mean (median, IQR)]	2.3 (1.0, 1.0–3.0)	0.6 (1.0, 0–1.0)	44 (3.0, 2.0–7.0)
PELOD-2 [mean (median, IQR)]	2.0 (2.0, 0–3.0)	0.6 (0, 0–2.0)	3.6 (3.5, 2.0–6.0)
System failure (*n*, %)^b^			
Cardiovascular dysfunction	14 (47)	3 (19)	11 (79)
Respiratory dysfunction	9 (30)	0	9 (64)
Hematalogic dysfunction	6 (20)	0	6 (43)
Neurologic dysfunction	5 (17)	0	5 (36)
Hepatic dysfunction	5 (17)	2 (13)	3 (21)
Renal dysfunction	5 (17)	0	5 (36)

^a^Among survivors

^b^System failure defined as pSOFA >  = 1 for system category(18)

### Characterization of respiratory failure (Table 2)

Among patients admitted to the PICU, NIPPV (bilevel or continuous positive pressure, HFNC) was required by 30% of patients; 6.7% were managed exclusively with HFNC.

Among PICU patients, 27% (*n* = 8) required endotracheal intubation. The average oxygen saturation index (OSI), a non-invasive measure of lung disease severity, upon intubation was 14. Median number of ventilator-free days was 15. One patient required veno-venous extracorporeal membranous oxygenation (4 days) for severe pARDS, and one patient was escalated to high-frequency percussive ventilation and eventually died secondary to refractory hypoventilation with severe hypercarbia and hypoxemia. This patient was evaluated for ECMO and deemed not to be a candidate based on institutional guidelines due to pre-existing comorbidities, irrespective of COVID-19 status.

### Complications, outcomes, and therapies (Table 2)

Two patients experienced secondary infections during their hospital course (peritonitis and bacterial pneumonia). No concurrent viral infections were documented in any patients.

For those discharged from the PICU (*n* = 29), median PICU length of stay was 3.6 days. At the close of data collection, 98% of the total cohort was discharged home and one child died (1.3%).

Interventions targeting COVID-19 among PICU patients included: hydroxychloroquine 63%, azithromycin 37%, corticosteroids 30%, remdesivir 27%, tocilizumab (anti-IL6R) 13%, anakinra (anti-IL1R) 13%, and intravenous immunoglobulin (IVIg) 10%.

### Factors associated with PICU admission and organ dysfunction (Table 3)

**Table 3:** Outcomes for PICU admission and organ dysfunction

	PICU admission *N* = 30	No PICU admission *N* = 47	*p *value
Outcome: PICU admission			
Age			0.0152
> = 12 years	20 (66.67)	18 (38.30)	
< 12 years	10 (33.33)	29 (61.70)	
Any comorbidity			0.2267
Yes	17 (56.67)	20 (42.55)	
No	13 (43.33)	27 (57.45)	
BMI category (*N* = 45)^a^			0.7310
Overweight/obese	7 (35.00)	10 (40.00)	
Underweight/normal weight	13 (65.00)	15 (60.00)	
Laboratory data			
Initial CRP (*N* = 37)			0.0053
Median (IQR)	54.10 (13.90, 161.90)	9.30 (4.00, 33.10)	
Initial ferritin (*N* = 42)			0.8495
Median (IQR)	446.05 (307.35, 809.35)	525.30 (160.20, 1003.00)	
Initial WBC (*N* = 69)			0.1455
Median (IQR)	7.8 (5.4–17.1)	7.8 (4.9–12.3)	
Initial platelets			0.0612
Median (IQR)	231 (182–206)	267 (200–419)	

	Organ dysfunction *N* = 14	No organ dysfunction *N* = 63	*p *value
Outcome: organ dysfunction			
Age			0.2166
> = 12 years	9 (64.29)	29 (46.03)	
< 12 years	5 (35.71)	34 (53.97)	
Any comorbidity			0.8719
Yes	7 (50.00)	30 (47.62)	
No	7 (50.00)	33 (52.38)	
BMI category (*N* = 45)^1^			0.7224
Overweight/obese	5 (45.45)	12 (35.29)	
Underweight/normal weight	6 (54.55)	22 (64.71)	
Laboratory data			
Initial CRP^c^			0.0011
Median (IQR)	109.70 (54.25, 218.15)	13.90 (4.50, 46.70)	
Initial ferritin^b^			0.2125
Median (IQR)	454.60 (373.70, 1039.00)	487.10 (178.90, 592.40)	
Initial WBC (*N* = 69)			0.0341
Median (IQR)	15.7 (6.4–18.5)	7.8 (4.8–11.4)	
Initial platelets			
Median (IQR)	149 (100–280)	268 (203–392)	0.0025

Organ dysfunction defined as pSOFA >  = 2, No organ dysfunction included floor patients and ICU patients with pSOFA < 2

^a^Patients < 2 years old excluded (*n* = 25) and *n* = 7 missing >  = 2 years old

^b^Ferritin collected for 42 patients

^c^CRP collected for 37 patients

Age ≥ 12 years was associated with the need for PICU admission (*p* = 0.015). Presence of any collected comorbidity was not associated with PICU admission (*p* = 0.227).

Median initial CRP value for floor patients was 9.30 mg/L and for PICU patients 54.1 mg/L (*n* = 37 patients, *p* = 0.005). Median initial WBC, platelet, and ferritin value showed no difference between floor and PICU patients.

Fourteen patients met criteria for organ dysfunction. Older age, underlying comorbidity, and BMI were not associated with organ dysfunction. Median initial WBC was higher in the organ dysfunction cohort (15.7 versus 7.8K/μL, *p* = 0.034). Median initial platelet count was lower in the organ dysfunction cohort (149 versus 268K/μL, *p* = 0.003); this association remained when analyzed for non-hematologic organ dysfunction. Median initial ferritin was similar in organ dysfunction patients (454.6 ng/mL) and non-organ dysfunction patients (487.1 ng/mL). Median initial CRP was higher in the organ dysfunction group than non-organ dysfunction group (109.7 versus 13.9 mg/L, *p* = 0.001).

## Discussion

The incidence of critical illness from COVID-19 and risk factors for severe disease in pediatrics are not yet well defined. Multicenter studies have described high rates of comorbid conditions in hospitalized children with COVID-19 [11, 12]. We studied 77 children with COVID-19 admitted to two of our hospitals over a 7-week period with the goal of determining clinical characteristics and factors associated with PICU admission and acute organ dysfunction.

In our cohort of pediatric COVID-19, 39% required intensive care and 18% developed organ dysfunction, indicating that symptomatic pediatric patients are at risk of critical illness. The most common indications for PICU admission were hypoxia and hemodynamic instability.

Our study found that age > 12 years is associated with PICU admission (*p* = 0.015) when compared to younger children, although this association was unadjusted. Comorbid pulmonary disease and immunocompromised status have been associated with more severe disease in pediatric patients with other strains of *Coronaviridae *[30]. Presence of any comorbidity and underlying obesity were not associated with the need for PICU admission or organ dysfunction. The rate of comorbidity in our PICU cohort (42.6%) and our organ dysfunction cohort (50%) were notably lower than two previously published studies that utilized reports of disease from many different sites [11, 12]. As all of our subjects are from the same health system, we may have avoided some testing and reporting bias that may have affected previous multicenter studies, as all patients were triaged and treated with the same protocols. Further, our data indicate that healthy children can suffer severe manifestations of COVID-19 at similar rates to those with chronic medical conditions. There is a need for further study regarding the impact of comorbidities on disease severity in pediatric COVID-19 and regarding the prevalence of symptomatic disease in pediatric populations.

Marked elevations in serum CRP and WBC were noted in our PICU cohort. Elevations in CRP may be predictive of mortality in pediatric severe sepsis and septic shock [24]. Elevated CRP was associated with mortality in an adult COVID-19 cohort from China and need for MV in a pediatric COVID-19 cohort [13, 23]. We identified WBC, platelet count, CRP and ferritin a priori as variables to examine in relation to need for PICU admission and development of organ dysfunction. PICU admission and organ dysfunction were both associated with increased CRP on hospital presentation. Elevated WBC and thrombocytopenia were associated with organ dysfunction in our cohort. Future research to clarify the predictive value of CRP, WBC, and thrombocytopenia is warranted.

Multiple targeted therapies were administered to our cohort of PICU patients. The heterogeneity in treatment approach highlights the need for randomized controlled trials to determine the optimal treatment for the varying phenotypes of COVID-19. Further risk stratification using biomarkers may allow for more efficient trials in the future that can target patients at risk for more severe disease.

Limitations of our study include its retrospective design and relatively small sample size due to this disease’s emerging nature. Multivariable analysis was not possible due to limited sample size and missing data. Comparisons herein are intended to develop hypotheses for future testing. Increasing sample size could allow for further definition of risk factors associated with severe disease and organ dysfunction and allow for refinement of predictive factors of severe disease. Seven factors were identified a priori and to maintain appropriate power and study design; additional laboratory differences could not be evaluated for statistical significance between groups. Other statistical comparisons between groups of patients could be an area of future research.

There were missing lab values, more prominently in the general floor cohort with a shorter LOS and lower disease severity. This skew in missing data is typical of retrospective studies, given healthier patients tend to have less clinical indication for laboratory studies. Despite this, the data suggests an association between elevations in the inflammatory markers discussed and presence of organ dysfunction. CRP, WBC, and thrombocytopenia may be useful tools in predicting patients who will develop organ dysfunction and is a vital area of future research. As the COVID-19 pandemic has overwhelmed healthcare systems, early markers of organ dysfunction could assist in triage decisions. Future studies will be able to include a larger cohort of patients and allow for refined study of the clinical characteristics of severe pediatric COVID-19.

## Conclusions

COVID-19 can cause severe, life-threatening disease in children. Older children are more likely to require PICU admission. Our cohort did not find an association between underlying comorbidity and critical illness or organ dysfunction. CRP, WBC, and platelet count may be useful markers of organ dysfunction, and further study is needed.

## Data Availability

The data that support the findings of this study are available on request from COVID19@northwell.edu.

## Abbreviations

BMI: Body mass index
CCMC: Cohen Children’s Medical Center
COVID-19: Coronavirus disease-2019
CRP: C-reactive protein
HFNC: High-flow nasal cannula
ICU: Intensive Care Unit
IQR: Interquartile range
LMV: Length of mechanical ventilation
LOS: Length of stay
MIS-C: Multisystem Inflammatory Syndrome in Children
MV: Mechanical ventilation
NIPPV: Non-invasive positive pressure ventilation
OSI: Oxygen saturation index
pARDS: Pediatric acute respiratory distress syndrome
PELOD-2: Pediatric Logistic Organ Dysfunction-2
PICU: Pediatric intensive care unit
pSOFA: Pediatric Sequential Organ Failure Assessment
SARS-2-CoV: Severe acute respiratory syndrome coronavirus 2
SIUH: Staten Island University Hospital
SOFA: Sequential Organ Failure Assessment
SpO2: Oxygen saturation
US: United States
WBC: White blood cell
